# Exposure to particle debris generated from passenger and truck tires induces different genotoxicity and inflammatory responses in the RAW 264.7 cell line

**DOI:** 10.1371/journal.pone.0222044

**Published:** 2019-09-10

**Authors:** Anna Poma, Giulia Vecchiotti, Sabrina Colafarina, Osvaldo Zarivi, Lorenzo Arrizza, Piero Di Carlo, Alessandra Di Cola

**Affiliations:** 1 Department of Life, Health and Environmental Sciences, University of L'Aquila, L'Aquila, Italy; 2 Center for Microscopy, University of L'Aquila, L'Aquila, Italy; 3 Department of Psychological, Health and Territorial Sciences, University "G. d'Annunzio" of Chieti-Pescara, Chieti, Italy; 4 Center of Excellence on Aging and Translational Medicine—Ce.S.I.—Me.T., University "G. d'Annunzio" of Chieti-Pescara, Chieti, Italy; 5 Tun Abdul Razak Research Centre, Brickendonbury, Hertford, United Kingdom; Consiglio Nazionale delle Ricerche, ITALY

## Abstract

A number of studies have shown variable grades of cytotoxicity and genotoxicity in *in vitro* cell cultures, laboratory animals and humans when directly exposed to particle debris generated from tires. However, no study has compared the effects of particles generated from passenger tires with the effects of particles from truck tires. The aim of this study was to investigate and relate the cyto- and genotoxic effects of different types of particles (PP, passenger tire particles vs. TP, truck tire particles) *in vitro* using the phagocytic cell line RAW 264.7 (mouse leukaemic monocyte macrophage cell line). The viability of RAW 264.7 cells was determined by the 3- (4,5-dimethylthiazol-2-yl) -5- (3-carboxymethoxyphenyl) -2- (4-sulfophenyl) -2H-tetrazolium (MTS) assay following exposure for 4, 24 and 48 hours to different particle concentrations (10 μg / ml, 25 μg / ml, 50 μg / ml, 100 μg / ml). The effects of particles of passenger and truck tires on cell proliferation and genotoxicity were evaluated by means of the cytokinesis-block micronucleus (CBMN) assay following exposure for 24 hours to different particle concentrations (10 μg / ml, 25 μg / ml, 50 μg / ml, 100 μg / ml). In MTS assay, after 24 hours, it was found that PP induced a 30% decrease in metabolic activity at a concentration of 10 μg/ml, while TP caused reductions of 20% and 10% at concentrations of 10 μg/ml and 50 μg/ml, respectively. At 48 hours after the treatments, we observed increased metabolic activity at 50 μg/ml and 100 μg/ml for the PP while only at 50 μg/ml for the TP. The CBMN assay showed a significant increase in the number of micronuclei in the cells incubated with PP in all experimental conditions, while the cells treated with TP showed a meaningful increase only at 10 μg /ml. We utilized the TNF-α ELISA mouse test to detect the production of tumour necrosis factor-alpha (TNF-α) in RAW 264.7 cells. The effect of passenger and truck particles on TNF-α release was evaluated following exposure for 4 and 24 hours.

After 4 hours of incubation, the cells treated with PP and TP at 100 μg / ml showed a slight but significant increase in TNF-α release, while there was a significant increase in the release of TNF-α after 24 hours of incubation with both tire samples in the cells treated with 50 and 100 μg / ml PP. The data obtained show a higher cytotoxic, clastogenic/genotoxic and inflammatory effects of passenger compared to the truck tire particles.

## Introduction

Air pollution is one of the most urgent environmental problems affecting human health. In recent decades, several studies have been conducted on air pollution and its direct and indirect consequences on human health and ecosystems.

In addition to such well-known air pollutants as ozone, sulfur oxides, nitrogen oxides, and volatile organic compounds (VOC), atmospheric particulate matter (PM) is a notable air pollutant. PM refers to all small microscopic liquid or solid particles suspended in the air with diameters between 3 nm and 30 μm, while particles with larger diameters (>30 μm) tend to deposit on the ground by gravity and are therefore no longer suspended in the atmosphere. The problem of the negative impact that the various constituents of atmospheric particulate have on human health is an increasingly researched topic. Specific epidemiological studies have indicated a link between high concentrations of atmospheric particles and increased admissions into hospitals and mortality rates, thereby demonstrating the negative effects on human health of short- and long-term exposure to environmental PM [1,2]. The adverse health effects of airborne particles include respiratory morbidity, which is manifested by reduced pulmonary function, cardiovascular morbidity, cancer, and death [3].

In this regard, it must be considered that one of the most significant contributors to atmospheric PM is car traffic, especially in urban areas; in fact, road traffic contributes more than 80% of the city's breathable PM_10_ [4].

It is common to consider vehicular traffic as a source of environmental pollution due to the release of such pollutants as hydrocarbons, nitrogen oxide, carbon, and all of the combustion exhausts released into the atmosphere; however, road vehicles are actually a more complex source of pollutants. It is imperative to consider that in addition to emissions from engine exhaust, dusts derived from tire wear are responsible for such health problems as asthma, allergies and cardiovascular problems.

The scientific community is increasingly interested in quantifying the influence that this component of the air we breathe every day, that is, tire particulates, exerts on environmental pollution.

We can introduce the concept of genotoxic risk associated with the emission of rubber powders from the rubber manufacturing industry [5]; the genotoxic risk associated with rubber is strongly influenced by the composition of the mixtures used (rubber formulation) and by the type of material processing. Therefore, *in vitro* studies were conducted to evaluate chromosomal damage, such as chromosomal aberrations and micronuclei frequency, on the DNA of exposed individuals. In particular, one study showed an increase in chromosomal aberration as an index of genotoxicity in exposed tire workers [6].

Based on this information, the effects of tire-wear-generated rubber particles on human health are being increasingly investigated.

Studies have been conducted in Japan that have allowed the volume of dust emitted per tire to be calculated. Taking into account the assumptions that the average life of a tire is approximately five years and because the depth of a tread differs from tire to tire, to estimate the wear, it was observed that in a five-year period, a total of 8.736.227 m^3^ (1.747.245 m^3^ per year) of rubber powder is produced from a single tire [7].

The typical values of material emission from cars tires fall within a range between 10 mg / km and 90 mg / km, but only approximately 4 or 6 mg / km are found as components of the atmospheric particulate. This finding suggests that generally between 1% and 15% of the tire wear material is emitted as atmospheric PM_10,_ while the remaining percentage is deposited onto the road surface Inizio modulo[8–9].

The general components of tires (which can vary from vehicle to vehicle) consists of a great variety of tire rubbers, including natural rubbers, butadiene rubber, SBR styrene rubber, neoprene rubber, isoprene and polysulfide, to which vulcanizing agents are added. Sulfur, tiazol, sulfenamide, selenium, tellurium, organic peroxides, nitrocompounds, and azocomposites are aimed at improving durability in terms of good state of the rubber, while zinc, calcium, lead, magnesium oxides and sulfur compounds are added as accelerators of the vulcanization process.

Natural rubber (NR) is a material mainly composed of cis-polyisoprene [(C_5_H_8_)_n_]. NR is collected as liquid latex from tapped rubber trees before undergoing processing; among the curing components, it is worth noting mercaptobenzothiazole, an organosulfur compound. Studies have identified this compound as a potential human carcinogen [10,11]. This compound becomes air-borne as a result of wear on car tires, and it can be inhaled [12].

Generally, in tires, the percentage of zinc oxide is 1.2% of the total composition with some variations according to the type of tire; a significant fraction of Zn is released in soil from the rubber matrix within 1 year, even if the increase in soil pH limits the mobilization of Zn [13]. In the mixture of natural and synthetic rubber, we also found micro carbon particles (BC) with diameters between 3 and 500 nm as a rubber reinforcing compound. Apart from the added chemicals that represent a clear health risk, the row material NR latex, itself being a natural product, is well known to be the causal agent for latex allergy. NR latex allergy can be common among health and rubber workers, but according to studies conducted on individuals living near busy roads, there may be a link between tire dust and allergic reactions, cases of rhinitis, conjunctivitis, urticaria, and bronchial asthma [14].

Various metals have been found in the rubber mixture, the quantity of which clearly varies according to the type of tire and the manufacturer [15].

The tire PM in question has been classified on the basis of the dimension, i.e., on the basis of the aerodynamic diameter of the particles, and some early studies defined a mean diameter of tire wear particles of approximately 20 μm, with few reaching a diameter of less than 3 μm [16]. Other studies have shown that PM also have emission particles smaller than 3 μm in diameter [17]. Current research focuses on the impact on human health by assessing their potential cytotoxic and genotoxic effects and as promoters of inflammation to determine whether small particles (10 μm aerodynamic diameter) are able to enter the respiratory tract. The differences in PM size are due to the mechanism that leads to PM production, i.e., the thermal degradation of the polymer of the tire and the volatilization of the extension oils together with the subsequent condensation of material essentially leads to the emission of ultrafine particles, while normal mechanical wear leads to the emission of larger particles [16]. Because rubber dust particles do not dissolve easily in water, once they are absorbed into the human body, they accumulate and hence affect human health. The coarse particles (2.5–10 μm) appear to correlate to acute respiratory symptoms and therefore to a greater release of inflammatory cytokines following inhalation, while those defined as fine (0.1–2.5 μm) and ultrafine (less than 0.1 μm) could have a role in the long-term effect on the respiratory and cardiovascular system.

Interestingly, it was observed that not only the size of the PM but also the qualitative properties, such as shape and chemical composition, influence the ability of the particles to induce toxicity and an inflammatory response on exposed subjects [18]. The coarse particles generated by a road simulator mimicked the effect of the wear of tires when in contact with two different types of road pavement. It was shown that it was possible to estimate the release of inflammatory cytokines IL-6, IL-8, IL-10 and TNFα in the culture medium by the human macrophages exposed to wear particles generated from studded tires and pavement [19]. Additionally, the type of tire certainly influences the level of wear and therefore the amount of PM_10_ particles produced; another parameter that influences the phenomenon of wear and tear is weather; in fact, the greatest wear and tear emissions are found in the winter dry season and in spring [20–21].The information on the chemical composition of the tread emissions was already provided by studies dating back to the 1990s, such as the research by Rogge et al. 1993 [22]. These studies first suggested that tires, despite being considered primarily a source of organic compounds, consist of approximately 13% inorganic material, which is derived from the various dyeing agents, accelerators and other constituent additives. Research has identified suchInizio modulo metals as Al, Ca, Cu, Fe, Ti, Zn [23], and more recent studies have found the presence of other metals in tire wear debris contributing to airborne metal loads, including Mn, Fe, Co, Ni, Cu, Cd and Pb [24].

However, since there are different sources for the latter elements related to road traffic, the use of these elements as potential indicators for tire wear is still not broad. Zinc and organic zinc represent approximately 1% by weight of the material from which the tire tread is made [25,26], and their release with tire wear has been recognized as a significant source of Zn in the environment. Zn represents a good indicator of wear particles of tires and, with the exception of vehicle engine oil, tires represent the only significant contribution of organic zinc in airborne particles, as it is generally found in the mixture of the tire's formulations as an accelerator; however, the chemical composition of the tire wear particles is also influenced by the interaction of the tire with the road surface during the abrasion phenomenon due to the heat and friction occurring during the process, as well as the incorporation of material from the road surface that will blend with the wear particles, thereby forming particles with different mineral contents depending on the type of flooring [23].

The aim of this work was to evaluate the cytotoxic, genotoxic effect and the induction of the inflammatory response by tires rubber particles (PP and TP) using the murine alveolar macrophage cell line RAW 264.7 to evaluate the environmental risk linked to tire wear particulate exposure. In this regard, the cyto-genotoxic potential of the tire particles was assessed by exposing the RAW 264.7 macrophage cell line to different PP and TP concentrations in the culture medium; following the treatment, we evaluated the viability and metabolic activity of the cells by the MTS assay test of cell proliferation. To estimate the genotoxic potential, we used the CBMN assay. To study the inflammatory response induction, we determined the TNFα released by the treated cells into the culture medium.

An eight-stage Anderson cascade impactor [27] with a pre separator stage to eliminate particles with aerodynamic diameter >10 μm was used to collect the tire particulates, and we carried out morphological analysis through scanning electron microscopy (SEM). The results obtained were associated with the microanalysis of the tire particles to obtain a chemical and semiquantitative characterization of the single elements that make up the rubber tire particles.

## Materials and methods

### Preparation of the tire rubber sample

For our study, we used a rubber sample from the fragmentation of passenger and truck tires. The tire samples were obtained from the "Tun Abdul Razak Research Centre (TARRC) UK" research centre artificially, re-proposing the phenomenon of abrasion between the tire and a road simulator track. “Passenger” tires are usually the most economical tires available on the market and do not guarantee long-lasting tread life of the tire itself, which rather quickly leads to wear. The “truck” tires are generally used on off-road surfaces and guarantee a better road holding.

To obtain tire dust, we used Tissuelyser II (Qiagen) and Andersen Impactor, which allowed us to select particles under 10 μm. Before fragmenting the rubber, the samples were frozen for 24 hours at -80°C in an Eppendorf tube. The next day inside the tubes, two tungsten beads were added, and the tubes were centrifuged in three cycles of 2 min each at a frequency of 25 Hz. This first operation allowed us to obtain a finely and homogeneously particulate rubber composed of particles of different sizes, with most of them being smaller than or equal to 10 μm.

After pulverizing the sample, we sieved it through the Andersen Impactor to select the particles according to their size. An eight-stage Andersen cascade impactor [27] with a preseparator stage to eliminate particles with aerodynamic diameters >10 μm was used to collect the particulate.

The Andersen impactor can be ideally related to the human respiratory system which, in turn, can be regarded as a PM dimensional classifier where only the finest, dangerous particles are able to pass the upper filtering districts and reach the alveolar region.

The operation of fractioning to the impactor was then repeated further by inserting an adhesive stab at the first stage. Through this stab, we recovered a small amount of rubber that was analysed by SEM for the rubber particle morphological analysis and microanalysis of the metallic content of our samples.

### Cell culture

The *in vitro* toxicological study was conducted in a phagocytic cell line murine, RAW 264.7 (mouse leukaemic monocyte macrophage cell line, cultures from ECACC (European Collection of Authenticated Cell Cultures) supplied by Sigma 85062803).

The macrophage cell line was grown in Dulbecco's modified Eagle's medium supplemented with 10% fetal bovine serum, 2 mM L-glutamine, penicillin (100 UI/ml) and streptomycin (100 μg/ml) and maintained at 37°C in a humidified atmosphere (95%) under 5% CO_2_. Cells were seeded at approximately 10,000 cells/cm^2^ and passaged every 3±4 days as necessary; they were finally removed from the plates with 0.05% trypsin-0.02% EDTA solution (all cell material was purchased from Sigma-Aldrich).

### Evaluation of the viability and metabolic activity: MTS Test

The viability of RAW 264.7 cells was determined by the MTS assay using a CellTiter Cell Proliferation Test Kit (Promega, Madison, MI, USA). The analysis was performed according to the manufacturer's protocol. The effect of passenger and truck tires on cell proliferation was assessed following exposure for 4, 24 and 48 hours at different concentrations (10 μg/ml, 25 μg/ml, 50 μg/ml, 100 μg/ml). Cells were seeded at 5,000 cells/cm^2^ and after 24 hours treated with different concentrations of rubber powder (10 μg/ml, 25 μg/ml, 50 μg/ml, 100 μg/ml) at the established times in a humidified incubator in a controlled atmosphere (5% CO_2_, 80% humidity, 37°C). Prior to cell treatment, rubber particle suspensions were subjected to 20 minutes in an ultrasonic bath to improve the homogeneous dispersion of the particles in the medium. Each experimental condition represents a technical triplicate, data refer to the mean and standard deviation of three independent experiments. Adequate positive controls (cells treated with 0.1% Triton-X-100) were performed with each series of experiments (4, 24 and 48 hours). Cell culture absorbance was measured at 490 nm, and cell proliferation was evaluated [28].

The supernatants stored at -80°C will be used for the subsequent TNFα analysis.

### Evaluation of the inflammatory response: TNF-α test

To detect a potential increase in the level of TNF-α in RAW 264.7 cells, we utilized the ELISA Mouse Kit (Thermo Scientific). The effect of passenger and truck tires on TNF-α release was evaluated after exposure to different concentrations (10 μg/ml, 25 μg/ml, 50 μg/ml, 100 μg/ml) for 4 and 24 hours. The supernatants obtained from the MTS test were used to measure the presence of TNF-α. We collected cell culture supernatants, centrifuged them at 1000 x g for 10 min to remove cell debris and particles, and stored them at -80°C. Adequate positive controls (cells treated with 0.1 μg/ml lipolysaccharide, LPS) were performed with each series of experiments (4 and 24 hours). We assayed TNF-α release in the supernatants using an ELISA kit according to the manufacturer’s instructions. The absorbance was read at 450 nm using a 96-well plate reader. The amount of TNF-α is determined by extrapolating OD values to TNFα concentrations using the standard curve (0–2500 pg/ml).

### Cytokinesis-block micronucleus (CBMN) assay

CBMN was carried out with slight modifications according to the protocol of Fenech [29] and OECD guidelines [30]. The cell line RAW 264.7 was seeded in each flask with 2.5 x 10^5^ cells/flask, and after 24 hours of culture, the cells were exposed to different concentrations (10 μg/ml, 25 μg/ml, 50 μg/ml, 100 μg/ml) of passenger and truck tires for 48 hours. Colchicine was used as a positive control at 5 μg/ml at the same treatment time. Cytochalasin B no later than 20 hours after stimulation by passenger and truck tires was added to cell cultures at a final concentration of 3 μg/ml.

Cells were harvested after additional 24 hours and centrifuged for 8 minutes at 1100 rpm; next, the supernatant was removed, and cells treated 1 min with 0.075 M KCl hypotonic solution. The cells were washed in PBS, resuspended (approximately 5 x 10^6^ cells/ml) and spread onto glass slides (20 μl of cell suspension per slide). After air-drying, the cells were fixed twice with methanol/glacial acetic acid (6:1) for 10 min and stained with 5% Giemsa solution for 5 min. All procedures were conducted at room temperature. After washing with distilled water, the slides were rapidly dried in xylene and mounted with Canadian balsam. By using a Leitz light microscope at 400x and 1000x magnification and following the criteria of Fenech [29] guidelines, 1000 binucleated cells were analysed for each treatment. Three biological replicates for each sample were used for CBMN analysis with three technical replicates (slides) each.

For each experimental condition, we calculated the Cytokinesis Block Proliferation Index (CBPI), to determine the frequency of mononuclear cells, bi- and multinucleated, using the following formula: [(N° mononucleated cells) + (2 x N° binucleated cells) + (3 x N° multinucleated cells)] / (total number of cells).

### Analysis of the tire rubber particles by SEM

For the morphological analysis and the microanalysis of the truck and passenger rubber particles, we used the SEM Zeiss Gemini 500 equipped with EDS (Energy Dispersive Spectroscopy) Oxford Inca Energy 250. The purpose of EDS microanalysis is to provide specific information on the composition of the sample elements.

We inserted a carbon tape in the zero stage of the impactor to recover sufficient quantities of truck and passenger tires. The carbon tape was mounted onto an SEM stub and then coated with a thin gold film by sputtering method. The SEM observations were carried out at different magnifications, and morphological analysis of the particles was performed simultaneously to obtain the EDS microanalysis of selected particles.

### Statistical analysis

Student's t test (unpaired) was applied for the statistical analysis of the data obtained from the tests to verify if the mean value of the treated conditions differs significantly from a reference value (control). For statistically significant values, * = p <0.05; ** = p <0.005; *** = p <0.0005.

The error bars represent the standard error of the mean. The data were analysed using the GraphPad Prism software, version 6.0 (© 1995–2015 GraphPad Software, Inc.). Three independent experiments have been performed for all assays applied.

## Results

### MTS assay

The cytotoxicity of the passenger and truck rubber particles was measured by the MTS assay cell viability test, which evaluates the metabolic activation of cultured cells, RAW 264.7, after treatment at different concentrations.

The test determines whether cells increase their metabolic activity, measuring the reduction of MTS by a formazan soluble in the culture medium, as MTS reduction occurs only in viable and metabolically active cells. Compared to control cells, the data show a slight effect is noticeable with the passenger rubber particle treatment at 25 μg/ml, which induces a visible reduction in cell viability, while the truck rubber particles induce a slight increase in absorbance at 100 μg/ml. After 24 hours, the absorbance values indicate that the passenger rubber particles at 10 μg/ml induce a significant decrease of the metabolic activity equal to 22%, while the truck particles cause a reduction of 20% and 8% at concentrations of 10 μg/ml and 50 μg/ml, respectively.

**Fig 1:**
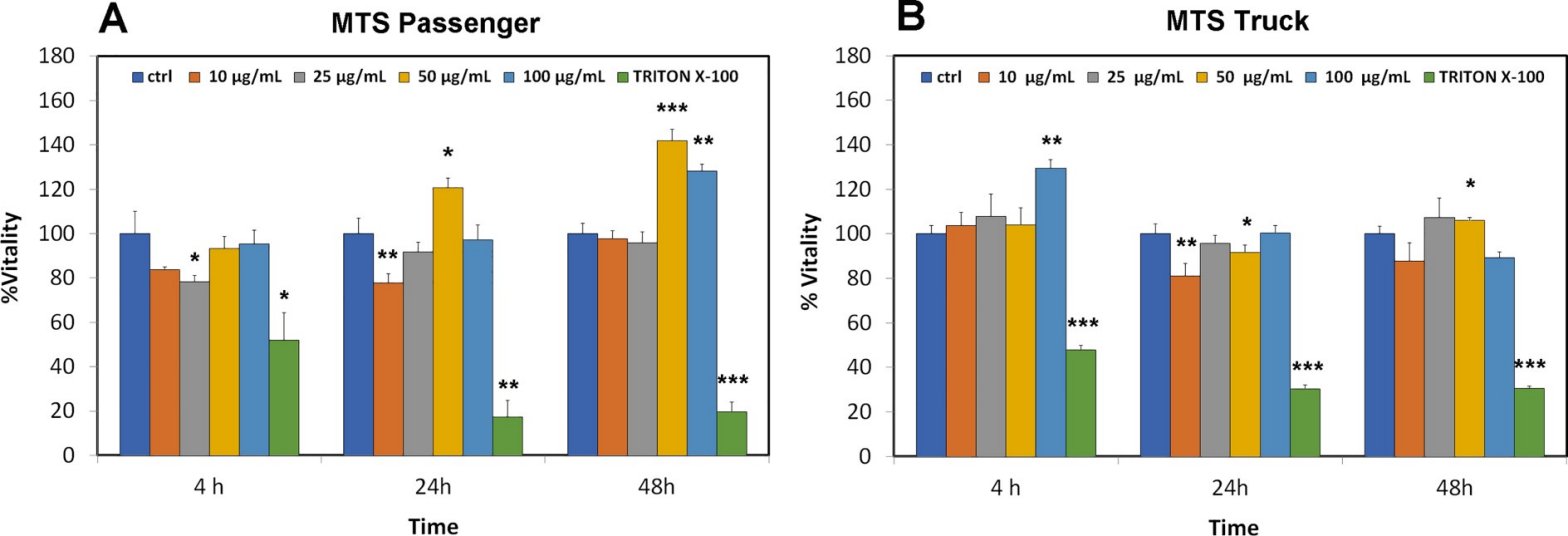
MTS test in RAW 264.7 cells. The effects of passenger (A) and truck (B) tires on cell proliferation were evaluated following exposures for 4, 24, 48 hours and compared to untreated cells. Triton-X-100 0.1% was used as a positive control. Significance values were determined according to Student’s t-test: * = p <0.05; ** = p <0.005; *** = p <0.0005; error bars represent the standard error of the mean.

At 48 hours after the treatments, we observed a significant increase in metabolic activity with 50 μg/ml and 100 μg/ml of the passenger tire particle treatment and at 50 μg/ml for the truck particles (Fig 1).

We can speculate that the observed viability trend, decreasing first after 24 hours treatment and increasing after 48 hours in both PP and TP treatments, may be observed because the cells first absorb and then act as macrophages. These results obtained with macrophagic *in vitro* cultured cells agree with the *in vivo* behaviour of alveolar macrophages, which, when in contact with a stimulus such as dust or bacteria are able to switch from an inactive to an active state [31]. Triton-X-100 0.1% was used as a positive control and it induced a significant viability decrease after 24 and 48 hours treatment in both PP and TP treatments (Fig 1). PM10 (coarse) airborne particulate matter reduced cell proliferation in RAW 264.7 cells as assessed by the MTT method [32] and it can be considered as a positive reference control in this work for particle uptake.

### Tests of micronuclei with block of cytodieresis with cytochalasin B “CBMN”

From the data obtained from the micronucleus test, we calculated the CBPI index "Cytokinesis Block Proliferation Index" to evaluate the cellular proliferation progression and therefore the cytostatic and cytotoxic effects followed by the different rubber particle treatments.

Compared to the control condition, the CBPI obtained from the cells incubated with the passenger particles increased in all experimental conditions, while in the cells treated with the truck tire particles, there was a slight but significant increase (p <0.027) only at 10 μg/ml.

**Fig 2:**
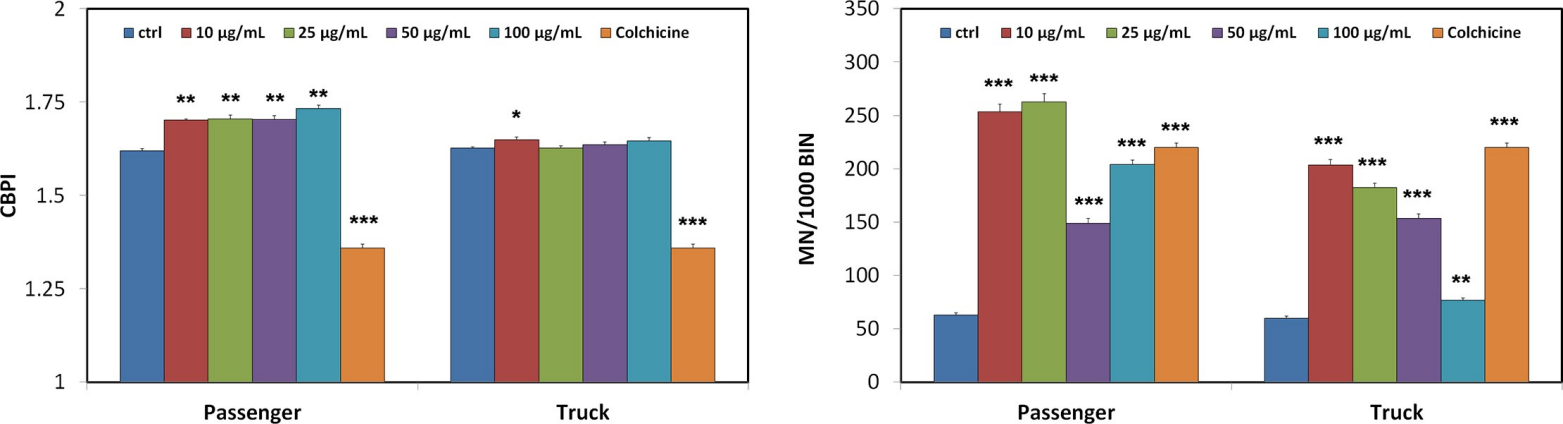
Micronuclei (BNMN) and CBPI index expression in the RAW 264.7 cells treated with particles from passenger tires and truck tires. CBPI index "Cytokinesis Block Proliferation Index" and micronuclei evaluated at the passenger and truck particle concentrations of 10, 25, 50 and 100 μg/ml. CBPI = ((N° mononucleated cells) + (2 x N° binucleated cells) + (3 x N° multinucleated cells)) / (total cell number). The numbers of micronuclei refer to 1000 binucleated cells. Colchicine was used as a positive control at 5 μg/ml at the same treatment time.

Regarding the induction of micronuclei (BNMN), we observed a significant increase in the formation of micronuclei at all concentrations of rubber powder, both in the cells treated with the passenger and in those treated with the truck (Fig 2).

### TNFα assay

**Fig 3:**
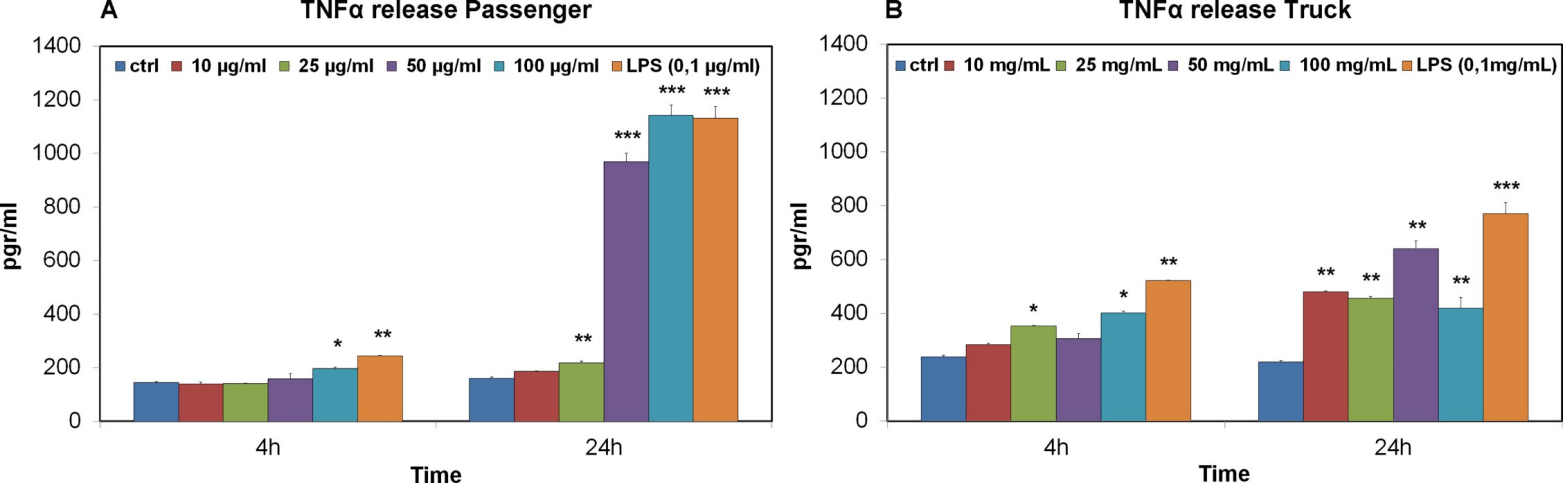
TNFα release in RAW 264.7 cells. The effects of passenger (A) and truck (B) tires on TNFα release were evaluated following exposure for 4 and 24 hours and compared to untreated cells. LPS (100 ng/ml) was used as a positive control. Significance values were determined according to Student’s t-test: * = p<0.05; ** = p<0.005; *** = p<0.0005; error bars represent the standard error of the mean.

The results show that the base level of TNFα in control cells after 4 and 24 hours of incubation is approximately 190 pg/ml. Data after 4 hours of incubation, for both passenger and truck particles, show a slight but significant increase in TNFα release compared to the control: at 100 μg/ml for the cells treated with passenger particles and at 25 and 100 μg/ml for the truck particles. A rather different result was obtained after 24 hours of incubation with both tire particle samples: we found a significant increase in the release of TNFα, in the cells treated with the 25, 50 and 100 μg/ml passenger tire particles with 200, 970 and 1142 pg/ml of released TNFα, respectively (Fig 3A). Regarding the treatment with the truck tire particles, the cytokine release was statistically significant at all concentrations. (Fig 3B).

We can also notice that at 24 hours how the truck particles compared to the passengers activate the macrophages already from the lowest concentration (10 μg/ml). In terms of the concentration of pro-inflammatory cytokines, we observed a greater release in the passenger, whereas truck particles showed a lower but early release that remained steadily low, even at higher concentrations of tire particles.

Interestingly, truck tire particles do not lead to the same toxic level effects that we have recorded for the passenger tires; this finding suggests that the higher percentage of natural rubber in truck tire formulations may be critical in reducing the toxic effects otherwise observed with the passenger tires. These findings, taken together, may have a significant positive impact on the natural rubber industry.

### Analysis of the rubber particles by SEM scanning electron microscopy

Passenger and truck particle samples were observed with SEM scanning electron microscopy equipped with EDS for elemental analysis.

**Fig 4:**
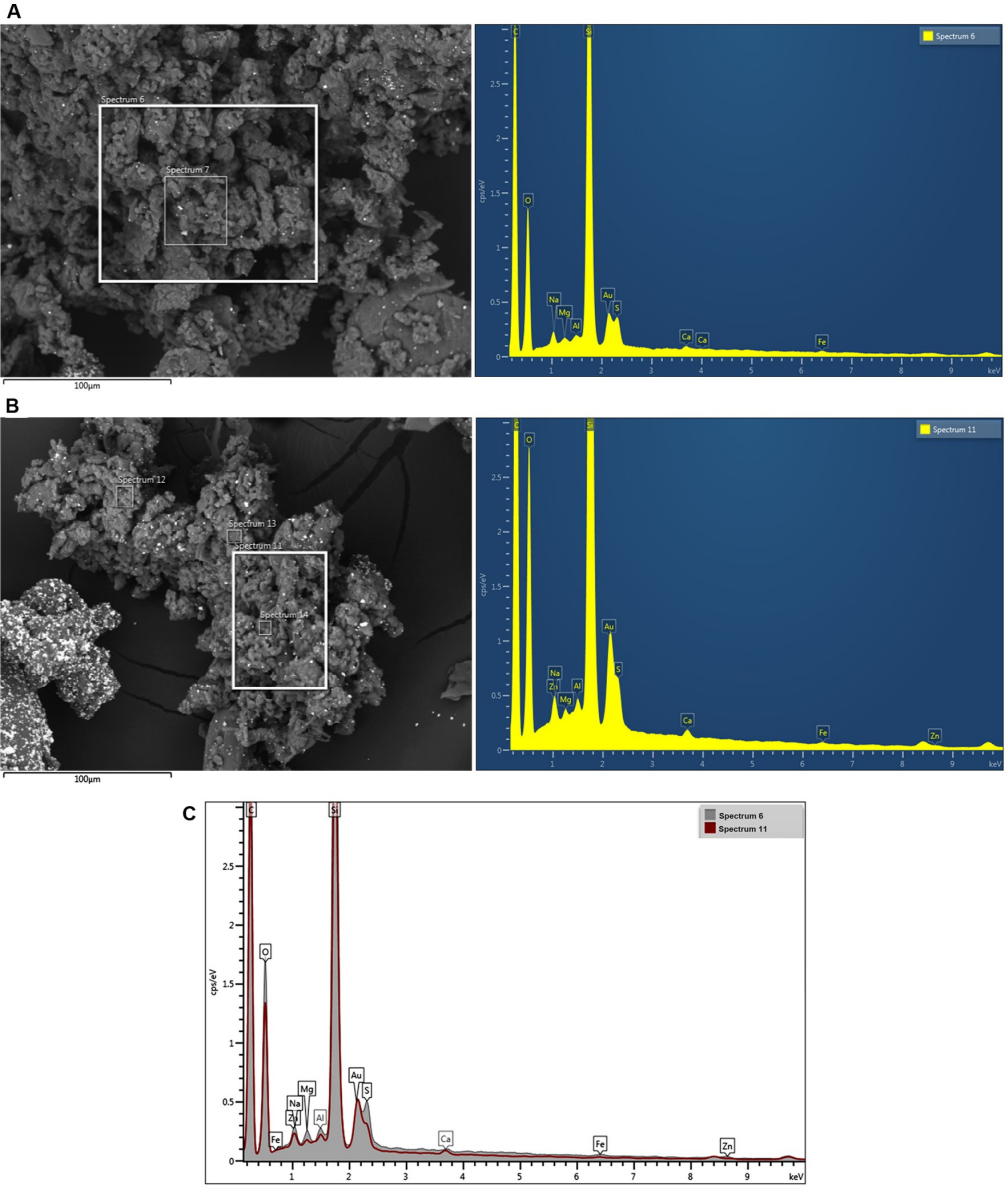
Scanning electron microscopy (SEM). Analysis of passenger (A) and truck (B) particles and the related spectrum of the microanalysis with EDS. (C) Comparison of EDS spectra of passenger and truck tire particles.

We focused on some significant particle samples recovered at the first stage of the impactor, of which we report the morphology images to verify their dimensions.

(Fig 4A and 4B) shows passenger and truck particles, respectively. At the morphological level, it is possible to notice a more coarse structure for the passenger particles (approximately 10–20 μm), while the truck particles appear to be larger than they actually are (approximately 1–2 μm) due to a strong tendency towards greater aggregation. In this regard, the truck tire particles are more friable than the passenger tire particles.

In addition to the morphological study, a compositional analysis was carried out using the EDS analysis to obtain an emission spectrum for particles (Fig 4A and 4B). In both spectrum particles (Fig 4C), different peaks corresponding to carbon and silica followed by oxygen may be observed; traces of sodium, magnesium, aluminium, calcium and iron are also present.

The most important difference between the truck and passenger spectra is the presence of sulfur and traces of zinc in passenger and truck tire particles, respectively.

The presence of gold in both passenger and truck tire particles is due to the metallization in sample preparation.

## Discussion

In this study, we approached the problem of the potential genotoxic, cytotoxic and pro-inflammatory effects of the particles produced as a result of tire wear, leading us to understand how these particles may become environmental pollutants of atmospheric aerosols. In previous studies, it has been observed that not only the size of the PM but also the qualitative properties, such as shape and chemical composition, influence the ability of tire rubber particles to induce an inflammatory response in exposed human subjects and cultured macrophages. Tire particles may exert harmful effects on health and/or the environment, attributable to the various components of the rubber used for the production of tires, to metals found in formulation mixtures that come into contact with our respiratory system or are deposited primarily onto the road surface [7–8, 18–20]. In this work, we have been able to see that both types of rubber particles (passenger and truck) have a biological effect *in vitro* estimated by the induction of genotoxicity, inflammatory response and cytotoxicity. From the statistically significant results obtained with both samples of rubber particles it is possible to notice that the passengers particles have induced higher production of proinflammatory cytokine (TNFα release), genotoxic/clastogenic response (BNMN formation) and activation of macrophages in MTS test. Genotoxic potential may be associated with chemical components: passenger particles consists mainly of synthetic polymers (about 30%) such as butadiene, halogenated polyisobutadiene and mercaptobenzothiazole, the latter identified as a potential human carcinogen. On the contrary the main component of the truck tires is natural rubber (34%), the main component of which is cis-polyisoprene, collected as latex from rubber trees (*Hevea brasiliensis*). Moreover truck tires generally contain more natural rubber and less carbon black than passenger car tires; carbon black is a major component of inhalable particulate matter directly emitted from the combustion of fossil fuels [33], and it is thought to mediate many of the adverse health effects reported in epidemiological studies to be associated with PM [34]. Passenger particles induced a genotoxic effect evidenced by micronuclei which increased at all concentrations while truck particles at the same concentration values showed a lower genotoxic response in terms of micronuclei induction.

In the tests that we carried out, there is no dose dependent response. The size and the chemical composition of particles are determinant for the cytotoxic, inflammatory and genotoxic activity in alveolar macrophages cell line. At the particles morphological level, it is possible to notice coarse structure for the passenger particles (approximately 10μm) while the truck particles appear to be larger due to a strong tendency towards aggregation which could be the reason of the not dose dependent effect observed, in particular at the concentrations of 50 and/or 100 μg/ml. In general the state of aggregation and the surface area of passenger and truck particles can change once they are introduced into the biological media as a result of surface-tension-mediated disaggregation of electrostatically or loosely agglomerated particulates.

The problem of environmental pollution due to wear particles is becoming increasingly urgent, and the most appropriate choice would be to reduce traffic and channel the technological development of ecological tires, improving tire composition and reducing wear and tear, which can contribute to lower emissions. Regarding the problem of lifting rubber dust deposited on the road surface, urban road washing has been demonstrated to reduce the impact on human health. It is also advisable to check environmental and health risk related to the exposure of tire wear particles by validated tests (i.e.OECD guidelines) aimed at accurately establishing the tire wear particles cytotoxic and genotoxic potential.

## Supporting information

S1 File Data MTS passenger. MTS test in RAW 264.7 cells treated with particles from passenger tires.
(PDF)

S2 File Data MTS truck. MTS test in RAW 264.7 cells treated with particles from truck tires.
(PDF)

S3 File Micronuclei passenger. Micronuclei (BNMN) and CBPI index expression in RAW 264.7 cells treated with particles from passenger tires.
(PDF)

S4 File Micronuclei truck. Micronuclei (BNMN) and CBPI index expression in RAW 264.7 cells treated with particles from truck tires.
(PDF)

S5 File TNF-alpha data passenger. TNF-α release in RAW 264.7 cells treated with passenger tires.
(PDF)

S6 File TNF-alpha data truck. TNF-α release in RAW 264.7 cells treated with truck tires.
(PDF)
