# Supplementary material for: Exposure to particle debris generated from passenger and truck tires induces different genotoxicity and inflammatory responses in the RAW 264.7 cell line
Source: PLoS One. 2019 Sep 10;14(9):e0222044. doi: 10.1371/journal.pone.0222044 (PMC6736306; doi:10.1371/journal.pone.0222044)
Supplement: S1 File — MTS test in RAW 264.7 cells treated with particles from passenger tires. (PDF) [file pone.0222044.s001.pdf]

Data MTS Test RAW 264.7 treated with passenger rubber

FOR EACH CONDITION THE SAMPLES HAVE BEEN MEDIATED

4 h

ctrl	10 µg/mL	25 µg/mL	50 µg/mL	100 µg/mL	TRITON X-100
1.087	0.903	0.757	1.237	0.926	0.467
0.865	0.895	0.831	1.153	0.796	0.743
0.883	0.837	0.870	1.119	0.886	0.699
0.808	0.865	0.903	0.989	0.850	0.281
1.133	0.890	0.731	1.014	1.136	0.550
1.510	0.880	0.802	0.830	0.806	0.523
		0.810	0.901	0.927	
		0.720	0.926	1.387	
		0.930	0.678	1.184	
		0.853	0.938	1.250	
				0.833	

24 h

ctrl	10 µg/mL	25 µg/mL	50 µg/mL	100 µg/mL	Triton X-100
1.869	1.007	1.099	1.672	1.838	0.352
1.659	1.006	1.500	1.916	1.911	0.338
1.398	1.238	1.413	1.979	1.770	0.309
1.088	1.315	1.607	2.155	1.526	0.224
1.487	1.083	1.347	1.816	1.734	0.219
1.520	1.124	1.360	1.749	1.488	0.234
	1.207	1.579	2.047	1.498	0.228
	1.383	1.520	1.990	1.887	0.231
		1.482	1.899	1.734	0.192
		1.652	1.441	1.143	
		1.583	1.303	0.884	
		1.401		1.028	
		0.877		0.913	
		0.939		1.082	
		1.353			

MEAN

	ctrl	10 mg/mL	25 mg/mL	50 mg/mL	100 mg/mL	Triton X-100
4 h	1.048	0.878	0.821	0.978	0.998	0.544
24h	1.504	1.170	1.381	1.815	1.460	0.259
48h	1.320	1.289	1.264	1.873	1.692	0.259
DS 4H	0.261	0.024	0.071	0.164	0.204	0.167
DS 24H	0.262	0.139	0.236	0.259	0.376	0.058
DS 48H	0.189	0.142	0.226	0.355	0.198	0.035
SE 4H	0.106	0.010	0.022	0.052	0.062	0.068
SE 24H	0.107	0.049	0.061	0.078	0.101	0.019
SE 48H	0.063	0.047	0.063	0.098	0.053	0.012

48 h

ctrl	10 µg/mL	25 µg/mL	50 µg/mL	100 µg/mL	Triton X-100
1.165	1.053	1.370	2.183	1.548	0.269
0.962	1.429	1.278	2.347	1.525	0.256
1.207	1.077	1.368	2.312	1.554	0.312
1.465	1.465	1.336	2.299	1.597	0.213
1.576	1.376	1.470	1.639	1.985	0.214
1.285	1.285	1.383	2.031	1.665	0.226
1.334	1.334	1.456	1.987	1.962	0.285
1.480	1.280	1.551	1.880	1.940	0.272
1.402	1.302	1.281	1.857	1.937	0.285
		1.226	1.546	1.817	
		0.974	1.379	1.709	
		0.927	1.450	1.439	
		0.807	1.435	1.477	
				1.526	

Data passenger MEAN expressed in percentage

MEAN

	ctrl	10 µg/mL	25 µg/mL	50 µg/mL	100 µg/mL	Triton X-100
4 h	1.048	0.878	0.821	0.978	0.998	0.544
24h	1.504	1.170	1.381	1.815	1.460	0.259
48h	1.320	1.289	1.264	1.873	1.692	0.259

MEAN PERCENTAGE

	ctrl	10 µg/mL	25 µg/mL	50 µg/mL	100 µg/mL	Triton X-100
4 h	100.000	83.827	78.349	93.402	95.294	51.893
24h	100.000	77.832	91.832	120.716	97.076	17.196
48h	100.000	97.678	95.752	141.909	128.177	19.638

Standard Error Percentage

	ctrl	10 µg/mL	25 µg/mL	50 µg/mL	100 µg/mL	Triton X-100
% SE 4H	10.166	1.126	2.723	5.291	6.172	12.531
% SE 24H	7.102	4.204	4.417	4.300	6.890	7.485
% SE 48H	4.766	3.667	4.956	5.253	3.134	4.455
