# Supplementary material for: Exposure to particle debris generated from passenger and truck tires induces different genotoxicity and inflammatory responses in the RAW 264.7 cell line
Source: PLoS One. 2019 Sep 10;14(9):e0222044. doi: 10.1371/journal.pone.0222044 (PMC6736306; doi:10.1371/journal.pone.0222044)
Supplement: S2 File — MTS test in RAW 264.7 cells treated with particles from truck tires. (PDF) [file pone.0222044.s002.pdf]

Data MTS Test RAW 264.7 treated with truck rubber

FOR EACH CONDITION THE SAMPLES HAVE BEEN MEDIATED

4 h

ctrl	10 µg/mL	25 µg/mL	50 µg/mL	100 µg/mL	TRITON X-100
0.852	0.947	1.072	0.998	1.161	0.446
1.004	0.953	0.854	1.202	1.537	0.474
0.960	0.870	1.206	1.284	1.310	0.506
1.055	1.167	1.568	1.047	1.231	0.502
1.087	1.084	0.945	1.056	1.259	0.458
1.099	1.265	0.887	0.709	1.337	0.506

24 h

ctrl	10 µg/mL	25 µg/mL	50 µg/mL	100 µg/mL	TRITON X-100
1.907	1.520	1.649	1.441	1.600	0.547
2.024	1.583	1.489	1.745	1.754	0.514
1.779	1.478	1.838	1.461	1.751	0.489
1.534	1.219	1.859	1.677	1.653	0.538
1.668	1.105	1.523	1.713	1.771	0.544
1.564	1.580	1.664	1.560	1.995	0.545

48 h

ctrl	10 µg/mL	25 µg/mL	50 µg/mL	100 µg/mL	TRITON X-100
1.661	1.059	1.807	1.490	1.471	0.570
2.000	1.614	1.164	1.801	1.694	0.550
1.890	1.841	2.246	2.417	1.701	0.562
1.880	1.951	2.191	2.186	1.665	0.538
1.591	1.597	2.160	2.007	1.629	0.557
1.795	1.443	2.046	1.569	1.489	0.536

DATA MEAN

	ctrl	10 µg/mL	25 µg/mL	50 µg/mL	100 µg/mL	TRITON X-100
**4h**	1.010	1.048	1.089	1.049	1.306	0.482
**24h**	1.746	1.414	1.670	1.600	1.754	0.529
**48h**	1.803	1.584	1.936	1.912	1.608	0.552
**DS 4H**	**0.093**	**0.151**	**0.268**	**0.198**	**0.129**	**0.026**
**DS 24H**	**0.194**	**0.202**	**0.154**	**0.131**	**0.136**	**0.023**
**DS 48H**	**0.153**	**0.315**	**0.409**	**0.360**	**0.103**	**0.014**
**SE 4H**	**0.038**	**0.061**	**0.110**	**0.081**	**0.053**	**0.011**
**SE 24H**	**0.079**	**0.083**	**0.063**	**0.054**	**0.055**	**0.010**
**SE 48H**	**0.063**	**0.129**	**0.167**	**0.147**	**0.042**	**0.006**

Data truck MEAN expressed in percentage

MEAN

	ctrl	10 µg/mL	25 µg/mL	50 µg/mL	100 µg/mL	TRITON X-100
4 h	1.010	1.048	1.089	1.049	1.306	0.482
24h	1.746	1.414	1.670	1.600	1.754	0.529
48h	1.803	1.584	1.936	1.912	1.608	0.552

MEAN PERCENTAGE

	ctrl	10 µg/mL	25 µg/mL	50 µg/mL	100 µg/mL	TRITON X-100
4 h	100.000	103.781	107.842	103.946	129.354	47.746
24h	100.000	80.995	95.666	91.609	100.458	30.325
48h	100.000	87.871	107.368	106.037	89.202	30.625

TANDARD ERROR PERCENTAG

	ctrl	10 µg/mL	25 µg/mL	50 µg/mL	100 µg/mL	TRITON X-100
% SE 4H	3.767	5.870	10.065	7.720	4.033	2.237
% SE 24H	4.539	5.845	3.767	3.346	3.162	1.803
% SE 48H	3.471	8.127	8.624	7.683	2.605	1.006
