# Supplementary material for: Exposure to particle debris generated from passenger and truck tires induces different genotoxicity and inflammatory responses in the RAW 264.7 cell line
Source: PLoS One. 2019 Sep 10;14(9):e0222044. doi: 10.1371/journal.pone.0222044 (PMC6736306; doi:10.1371/journal.pone.0222044)
Supplement: S3 File — Micronuclei (BNMN) and CBPI index expression in RAW 264.7 cells treated with particles from passenger tires. (PDF) [file pone.0222044.s003.pdf]

DATA TEST MICRONUCLEI

CONDITIONS			CELLS MONON	CELLS BIN.	CELLS MULTIN.	TOTAL CELLS		CBPI	Micronuclei
ctrl -	Sample 1	GLASS 1	591	1000	1	1592		1.629396985	59
		GLASS 2	691	1000	1	1692		1.592198582	68
		GLASS 3	611	1000	1	1612		1.621588089	71
	Sample 2	GLASS 1	631	1000	3	1634		1.615667075	57
		GLASS 2	544	1000	5	1549		1.65203357	61
		GLASS 3	637	1000	2	1639		1.612568639	61
	Sample 3	GLASS 1	661	1000	4	1665		1.605405405	71
		GLASS 2	646	1000	2	1648		1.609223301	61
		GLASS 3	564	1000	1	1565		1.640255591	56
		MEAN	619.555556	1000	2.222222222	1621.777778		1.619815249	62.77777778
							SD	0.018381425	5.761462006
							SE	0.006127142	1.920487335
10 µg/mL	Sample 1	GLASS 1	532	1000	32	1564		1.680306905	223
		GLASS 2	506	1000	45	1551		1.702772405	261
		GLASS 3	507	1000	46	1553		1.703155184	257
	Sample 2	GLASS 1	502	1000	45	1547		1.704589528	245
		GLASS 2	476	1000	36	1512		1.708994709	289
		GLASS 3	495	1000	43	1538		1.706111834	255
	Sample 3	GLASS 1	480	1000	35	1515		1.706270627	259
		GLASS 2	487	1000	28	1515		1.697029703	221
		GLASS 3	500	1000	54	1554		1.712998713	270
		MEAN	498.333333	1000	40.44444444	1538.777778		1.702469956	253.3333333
							SD	0.008863071	21.48255106
							SE	0.002954357	7.160850353
25 µg/mL	Sample 1	GLASS 1	635	1000	165	1800		1.738888889	220
		GLASS 2	570	1000	130	1700		1.741176471	265
		GLASS 3	685	1000	125	1810		1.690607735	220
	Sample 2	GLASS 1	702	1000	165	1867		1.712372791	278
		GLASS 2	632	1000	134	1766		1.718006795	317
		GLASS 3	757	1000	172	1929		1.696734059	299
	Sample 3	GLASS 1	668	1000	154	1822		1.717892426	239
		GLASS 2	697	1000	121	1818		1.683168317	265
		GLASS 3	738	1000	97	1835		1.650681199	261
		MEAN	676	1000	140.3333333	1816.333333		1.705503187	262.6666667
							SD	0.028607871	33.01893396
							SE	0.009535957	11.00631132
50 µg/mL	Sample 1	GLASS 1	589	1000	79	1668		1.694244604	185
		GLASS 2	576	1000	62	1638		1.686202686	122
		GLASS 3	593	1000	78	1671		1.691801317	128
	Sample 2	GLASS 1	573	1000	62	1635		1.687461774	142
		GLASS 2	581	1000	49	1630		1.673619632	121
		GLASS 3	613	1000	69	1682		1.676575505	151
	Sample 3	GLASS 1	571	1000	125	1696		1.737028302	173
		GLASS 2	531	1000	133	1664		1.760817308	146
		GLASS 3	569	1000	111	1680		1.727380952	173
		MEAN	577.333333	1000	85.33333333	1662.666667		1.703903564	149
							SD	0.030362318	23.62202362
							SE	0.010120773	7.874007874
100 µg/mL	Sample 1	GLASS 1	543	1000	142	1685		1.762017804	221
		GLASS 2	625	1000	156	1781		1.736664795	198
		GLASS 3	521	1000	146	1667		1.775044991	184
	Sample 2	GLASS 1	578	1000	132	1710		1.739181287	201
		GLASS 2	537	1000	121	1658		1.749095296	189
		GLASS 3	601	1000	110	1711		1.713033314	198
	Sample 3	GLASS 1	596	1000	81	1677		1.692903995	217
		GLASS 2	535	1000	91	1626		1.726937269	223
		GLASS 3	612	1000	95	1707		1.697129467	205
		MEAN	572	1000	119.3333333	1691.333333		1.732445358	204
							SD	0.027945378	13.81122732
							SE	0.009315126	4.603742439

ctrl +	Sample 1	GLASS 1	899	537	8	1444	1.382963989	243
		GLASS 2	935	612	8	1555	1.403858521	222
		GLASS 3	867	432	11	1310	1.346564885	225
	Sample 2	GLASS 1	981	587	4	1572	1.378498728	213
		GLASS 2	865	435	5	1305	1.340996169	234
		GLASS 3	973	402	6	1381	1.299782766	213
	Sample 3	GLASS 1	871	439	11	1321	1.348978047	206
		GLASS 2	986	509	8	1503	1.349301397	214
		GLASS 3	1011	608	8	1627	1.383527966	210
						MEA	1.35938583	220
						SD	0.031118713	12.16552506
						SEM	0.010372904	4.05517502

	ctrl	10 µg/mL	25 µg/mL	50 µg/mL	100 µg/mL
CBPI	1.619815	1.70247	1.70550319	1.703903564	1.732445358
SEM	0.006127	0.002954	0.00953596	0.010120773	0.009315126

	ctrl	10 µg/mL	25 µg/mL	50 µg/mL	100 µg/mL
MN/1000 BIN	62.77778	253.3333	262.666667	149	204
SEM	1.920487	7.16085	7.87400787	4.603742439	4.05517502
