# Supplementary material for: Exposure to particle debris generated from passenger and truck tires induces different genotoxicity and inflammatory responses in the RAW 264.7 cell line
Source: PLoS One. 2019 Sep 10;14(9):e0222044. doi: 10.1371/journal.pone.0222044 (PMC6736306; doi:10.1371/journal.pone.0222044)
Supplement: S5 File — TNF-α release in RAW 264.7 cells treated with passenger tires. (PDF) [file pone.0222044.s005.pdf]

TNFa TEST Passenger

FOR EACH CONDITION THE SAMPLES HAVE BEEN MEDIATED

4 h lecture

CONDITIONS	ctrl	10 µg/ml	25 µg/ml	50 µg/ml	100 µg/ml	LPS (0,1 µg/ml)
	0.088	0.081	0.081	0.111	0.119	0.134
	0.096	0.086	0.082	0.118	0.13	0.139
	0.085	0.089	0.092	0.108	0.103	0.141
MEAN	0.090	0.085	0.085	0.112	0.117	0.138
	0.082	0.092	0.079	0.093	0.101	0.136
	0.085	0.096	0.089	0.089	0.110	0.131
	0.079	0.080	0.083	0.087	0.109	0.139
MEAN	0.082	0.089	0.084	0.090	0.107	0.135
	0.089	0.074	0.089	0.083	0.118	0.140
	0.085	0.079	0.084	0.085	0.112	0.132
	0.091	0.086	0.087	0.076	0.115	0.138
MEAN	0.088	0.080	0.087	0.081	0.115	0.137

Standard curve equation used to calculate the pgr x=(y-0,0147)/0,0005

4h (pg)

CONDITIONS	ctrl	10 µg/ml	25 µg/ml	50 µg/ml	100 µg/ml	LPS (0,1 µg/ml)
	149.933	141.267	140.600	195.267	205.267	246.600
	134.600	149.267	137.933	149.933	183.933	241.267
	147.267	129.933	143.933	133.267	200.600	243.933
MEAN	143.933	140.156	140.822	159.489	196.600	243.933
SD	8.192	9.714	3.006	32.086	11.215	2.667
ES	4.730	5.609	1.736	18.525	6.475	1.540

24 h lecture

CONDITIONS	ctrl	10 µg/ml	25 µg/ml	50 µg/ml	100 µg/ml	LPS (0,1 µg/ml
	0.092	0.106	0.131	0.495	0.570	0.534
	0.089	0.101	0.125	0.483	0.615	0.542
	0.097	0.114	0.130	0.492	0.637	0.565
MEAN	0.093	0.107	0.129	0.490	0.607	0.547
	0.093	0.112	0.119	0.536	0.587	0.569
	0.097	0.111	0.131	0.513	0.578	0.582
	0.082	0.107	0.124	0.538	0.644	0.570
MEAN	0.091	0.110	0.125	0.529	0.603	0.574
	0.099	0.108	0.128	0.489	0.553	0.628
	0.085	0.121	0.121	0.467	0.542	0.612
	0.119	0.089	0.103	0.481	0.546	0.623
MEAN	0.101	0.106	0.117	0.479	0.547	0.621

24h (pg)

CONDITIONS	ctrl	10 µg/ml	25 µg/ml	50 µg/ml	100 µg/ml	LPS (0,1 µg/ml)
	155.933	184.600	227.933	950.600	1185.267	1064.600
	151.933	190.600	219.933	1028.600	1176.600	1117.933
	172.600	182.600	205.267	928.600	1064.600	1212.600
MEAN	160.156	185.933	217.711	969.267	1142.156	1131.711
DS	10.961	4.163	11.496	52.548	67.305	74.956
ES	6.328	2.404	6.637	30.339	38.858	43.276

DATA HISTOGRAM

	ctrl	10 µg/ml	25 µg/ml	50 µg/ml	100 µg/ml	LPS (0,1 µg/ml)
4h	143.933	140.156	140.822	159.489	196.600	243.933
24h	160.156	185.933	217.711	969.267	1142.156	1131.711
ES	4.730	5.609	1.736	18.525	6.475	1.540
ES	6.328	2.404	6.637	30.339	38.858	43.276
