# Supplementary material for: Exposure to particle debris generated from passenger and truck tires induces different genotoxicity and inflammatory responses in the RAW 264.7 cell line
Source: PLoS One. 2019 Sep 10;14(9):e0222044. doi: 10.1371/journal.pone.0222044 (PMC6736306; doi:10.1371/journal.pone.0222044)

DATA TEST MICROCLEI TRUCK

| CONDITIONS | | | CELLS MONON. | CELLS BIN. | CELLS MULTIN. | TOTAL CELLS | | CBPI | Micronuclei |
|---|---|---|---|---|---|---|---|---|---|
| ctrl - | Sample 1 | GLASS 1 | 631 | 1000 | 2 | 1633 | | 1.614819 | 65 |
| | | GLASS 2 | 564 | 1000 | 3 | 1567 | | 1.641991 | 54 |
| | | GLASS 3 | 641 | 1000 | 4 | 1645 | | 1.612766 | 61 |
| | Sample 2 | GLASS 1 | 618 | 1000 | 0 | 1618 | | 1.618047 | 56 |
| | | GLASS 2 | 593 | 1000 | 1 | 1594 | | 1.628607 | 66 |
| | | GLASS 3 | 598 | 1000 | 5 | 1603 | | 1.630069 | 58 |
| | Sample 3 | GLASS 1 | 599 | 1000 | 6 | 1605 | | 1.63053 | 66 |
| | | GLASS 2 | 606 | 1000 | 4 | 1610 | | 1.626087 | 52 |
| | | GLASS 3 | 577 | 1000 | 2 | 1579 | | 1.635845 | 62 |
| | | | | | | | MEAN | 1.626529 | 60 |
| | | | | | | | SD | 0.00973 | 5.267826876 |
| | | | | | | | SEM | 0.003243 | 1.755942292 |
| 10 µg/mL | Sample 1 | GLASS 1 | 572 | 1000 | 8 | 1580 | | 1.643038 | 171 |
| | | GLASS 2 | 578 | 1000 | 9 | 1587 | | 1.641462 | 198 |
| | | GLASS 3 | 458 | 1000 | 5 | 1463 | | 1.690362 | 201 |
| | Sample 2 | GLASS 1 | 621 | 1000 | 7 | 1628 | | 1.62285 | 198 |
| | | GLASS 2 | 652 | 1000 | 11 | 1663 | | 1.614552 | 203 |
| | | GLASS 3 | 537 | 1000 | 5 | 1542 | | 1.654994 | 199 |
| | Sample 3 | GLASS 1 | 509 | 1000 | 7 | 1516 | | 1.668865 | 216 |
| | | GLASS 2 | 518 | 1000 | 4 | 1522 | | 1.662286 | 226 |
| | | GLASS 3 | 584 | 1000 | 9 | 1593 | | 1.639046 | 218 |
| | | | | | | | MEAN | 1.648606 | 203.3333333 |
| | | | | | | | SD | 0.023395 | 15.87450787 |
| | | | | | | | SEM | 0.007798 | 5.291502622 |
| 25 µg/mL | Sample 1 | GLASS 1 | 630 | 1000 | 7 | 1637 | | 1.619426 | 178 |
| | | GLASS 2 | 610 | 1000 | 9 | 1619 | | 1.628783 | 198 |
| | | GLASS 3 | 566 | 1000 | 11 | 1577 | | 1.648066 | 188 |
| | Sample 2 | GLASS 1 | 682 | 1000 | 5 | 1687 | | 1.598696 | 168 |
| | | GLASS 2 | 572 | 1000 | 10 | 1582 | | 1.644753 | 174 |
| | | GLASS 3 | 589 | 1000 | 9 | 1598 | | 1.637046 | 168 |
| | Sample 3 | GLASS 1 | 678 | 1000 | 6 | 1684 | | 1.60095 | 178 |
| | | GLASS 2 | 589 | 1000 | 9 | 1598 | | 1.637046 | 187 |
| | | GLASS 3 | 627 | 1000 | 9 | 1636 | | 1.622249 | 202 |
| | | | | | | | MEAN | 1.626335 | 182.3333333 |
| | | | | | | | SD | 0.017747 | 12.26784415 |
| | | | | | | | SEM | 0.005916 | 4.089281382 |
| 50 µg/mL | Sample 1 | GLASS 1 | 693 | 1000 | 5 | 1698 | | 1.594817 | 154 |
| | | GLASS 2 | 581 | 1000 | 8 | 1589 | | 1.639396 | 165 |
| | | GLASS 3 | 618 | 1000 | 8 | 1626 | | 1.624846 | 161 |
| | Sample 2 | GLASS 1 | 598 | 1000 | 3 | 1601 | | 1.628357 | 176 |
| | | GLASS 2 | 561 | 1000 | 5 | 1566 | | 1.644955 | 158 |
| | | GLASS 3 | 494 | 1000 | 4 | 1498 | | 1.672897 | 146 |
| | Sample 3 | GLASS 1 | 591 | 1000 | 4 | 1595 | | 1.631975 | 136 |
| | | GLASS 2 | 547 | 1000 | 1 | 1548 | | 1.647287 | 145 |
| | | GLASS 3 | 572 | 1000 | 4 | 1576 | | 1.639594 | 139 |
| | | | | | | | MEAN | 1.636014 | 153.3333333 |
| | | | | | | | SD | 0.020882 | 13.03840481 |
| | | | | | | | SEM | 0.006961 | 4.346134937 |
| 100 µg/mL | Sample 1 | GLASS 1 | 656 | 1000 | 2 | 1658 | | 1.605549 | 76 |
| | | GLASS 2 | 578 | 1000 | 7 | 1585 | | 1.639748 | 82 |
| | | GLASS 3 | 587 | 1000 | 2 | 1589 | | 1.631844 | 82 |
| | Sample 2 | GLASS 1 | 510 | 1000 | 2 | 1512 | | 1.664021 | 65 |
| | | GLASS 2 | 438 | 1000 | 6 | 1444 | | 1.700831 | 72 |
| | | GLASS 3 | 529 | 1000 | 4 | 1533 | | 1.657534 | 73 |
| | Sample 3 | GLASS 1 | 593 | 1000 | 1 | 1594 | | 1.628607 | 80 |
| | | GLASS 2 | 543 | 1000 | 3 | 1546 | | 1.650712 | 77 |
| | | GLASS 3 | 596 | 1000 | 2 | 1598 | | 1.628285 | 83 |
| | | | | | | | MEAN | 1.645237 | 76.66666667 |
| | | | | | | | SD | 0.027325 | 5.916079783 |
| | | | | | | | SEM | 0.009108 | 1.972026594 |

| | | | | | | | | | |
|---|---|---|---|---|---|---|---|---|---|
| ctrl + | Sample 1 | GLASS 1 | 899 | 537 | 8 | 1444 | | 1.382964 | 243 |
| | | GLASS 2 | 935 | 612 | 8 | 1555 | | 1.403859 | 222 |
| | | GLASS 3 | 867 | 432 | 11 | 1310 | | 1.346565 | 225 |
| | Sample 2 | GLASS 1 | 981 | 587 | 4 | 1572 | | 1.378499 | 213 |
| | | GLASS 2 | 865 | 435 | 5 | 1305 | | 1.340996 | 234 |
| | | GLASS 3 | 973 | 402 | 6 | 1381 | | 1.299783 | 213 |
| | Sample 3 | GLASS 1 | 871 | 439 | 11 | 1321 | | 1.348978 | 206 |
| | | GLASS 2 | 986 | 509 | 8 | 1503 | | 1.349301 | 214 |
| | | GLASS 3 | 1011 | 608 | 8 | 1627 | | 1.383528 | 210 |
| | | | | | | | MEAN | 1.359386 | 220 |
| | | | | | | | SD | 0.031119 | 12.16552506 |
| | | | | | | | SEM | 0.010373 | 4.05517502 |

| | ctrl | 10 µg/m | 25 µg/mL | 50 µg/mL | 100 µg/mL |
|---|---|---|---|---|---|
| **CBPI** | 1.626529 | 1.6486 | 1.62633516 | 1.6360139 | 1.645236778 |
| **SEM** | 0.003243 | 0.0078 | 0.005915696 | 0.0069607 | 0.009108372 |

| | ctrl | 10 µg/m | 25 µg/mL | 50 µg/mL | 100 µg/mL |
|---|---|---|---|---|---|
| **MN/1000** | 60 | 203.33 | 182.3333333 | 153.33333 | 76.66666667 |
| **SEM** | 1.755942 | 5.2915 | 4.089281382 | 4.3461349 | 1.972026594 |

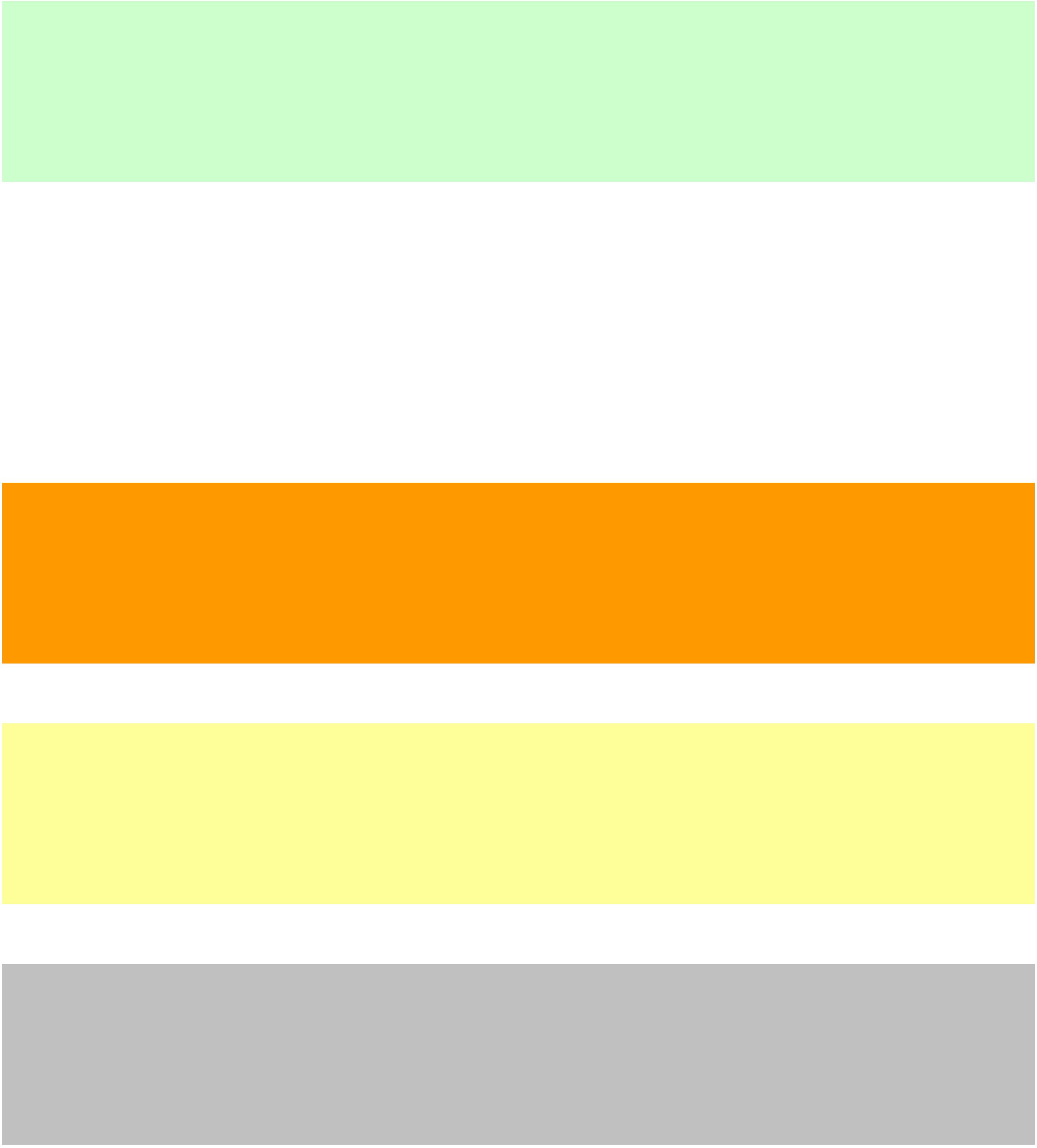

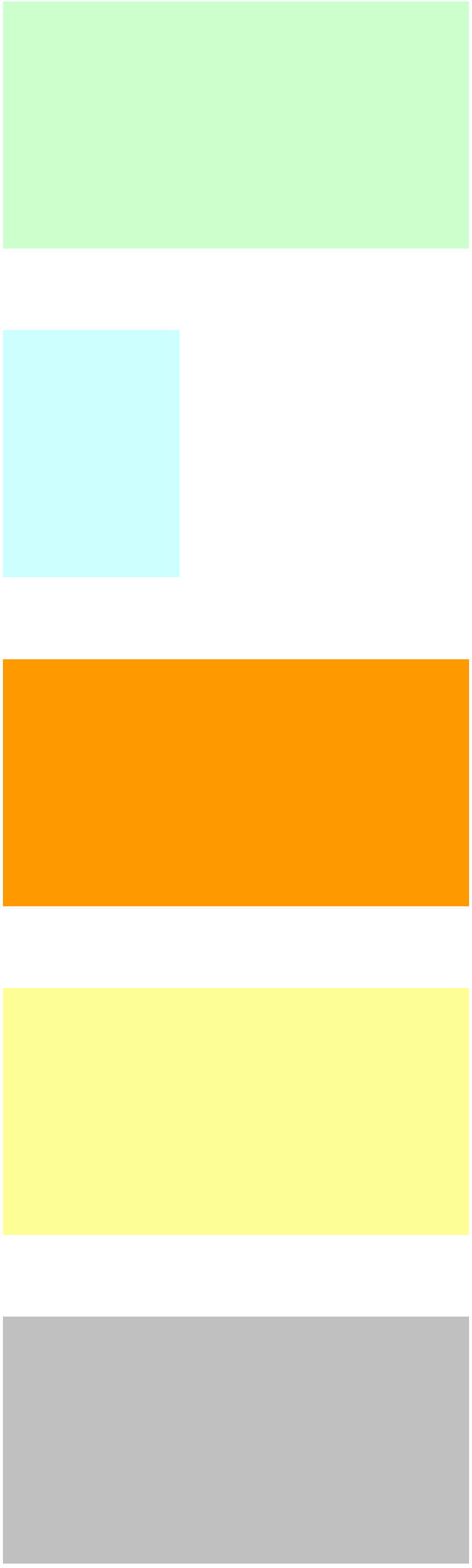

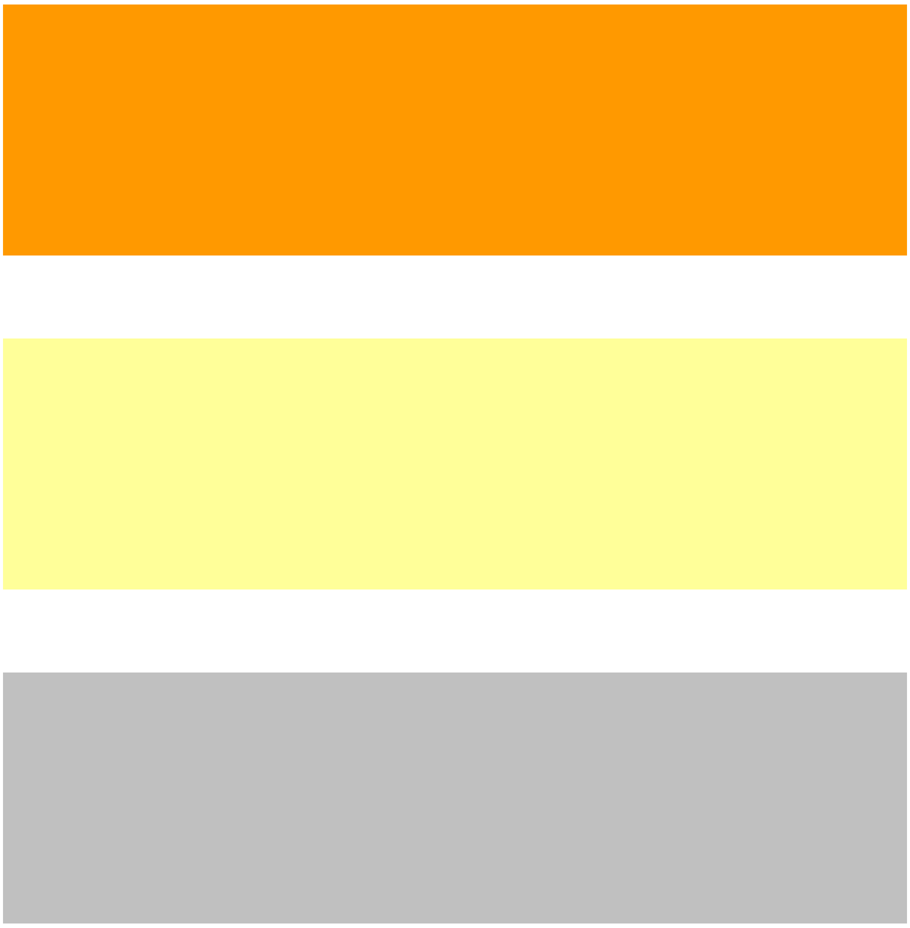

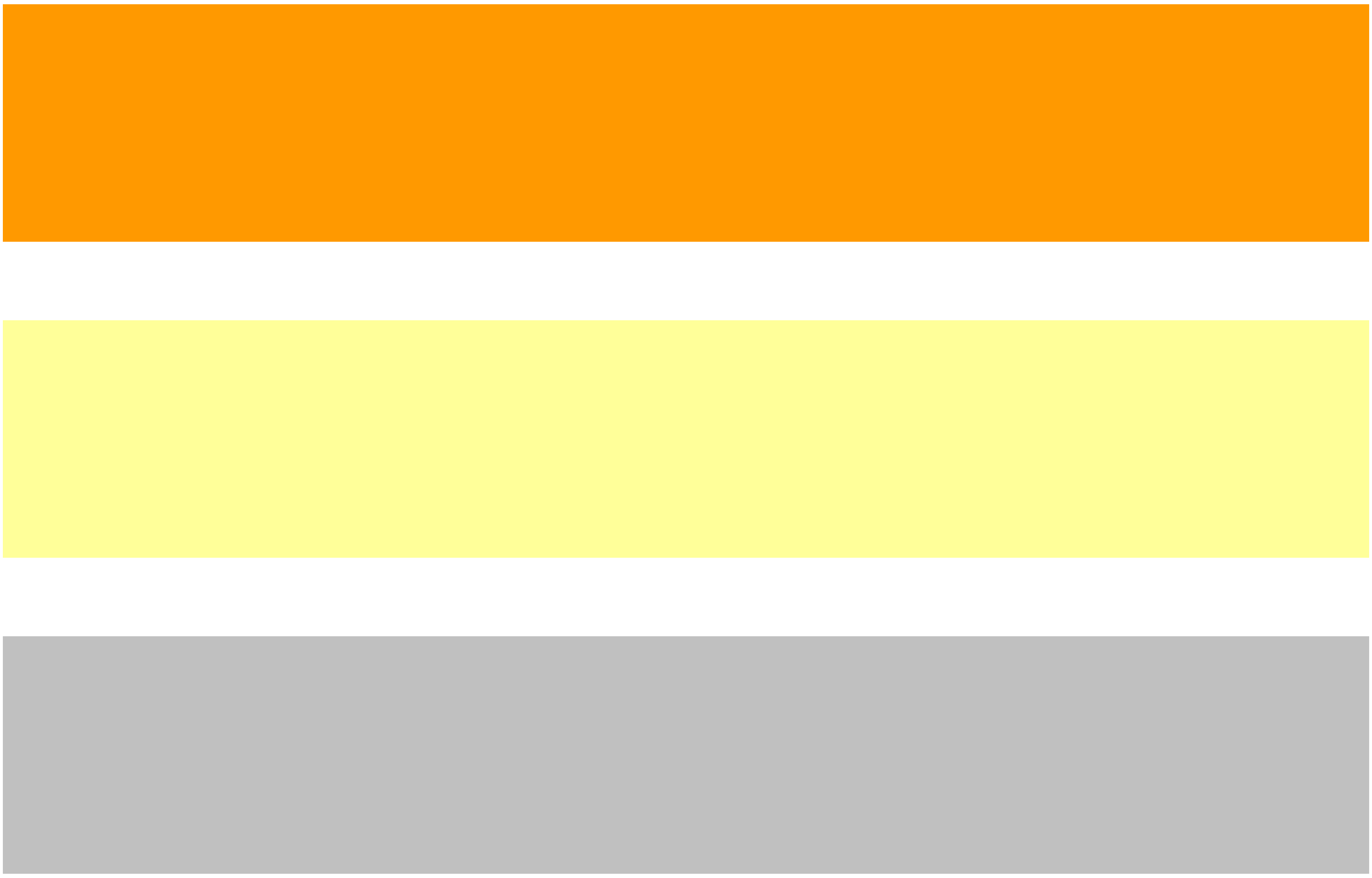

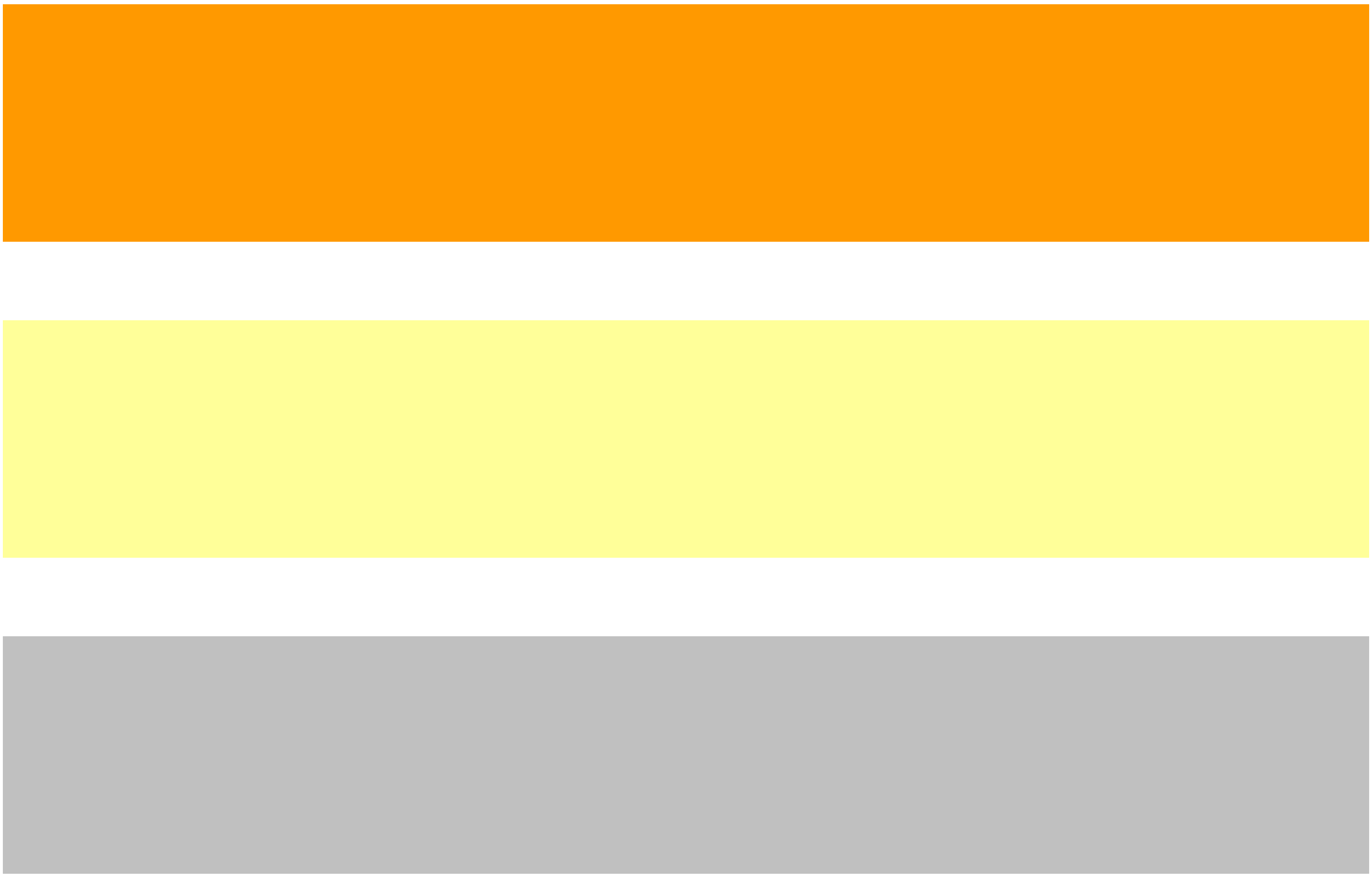

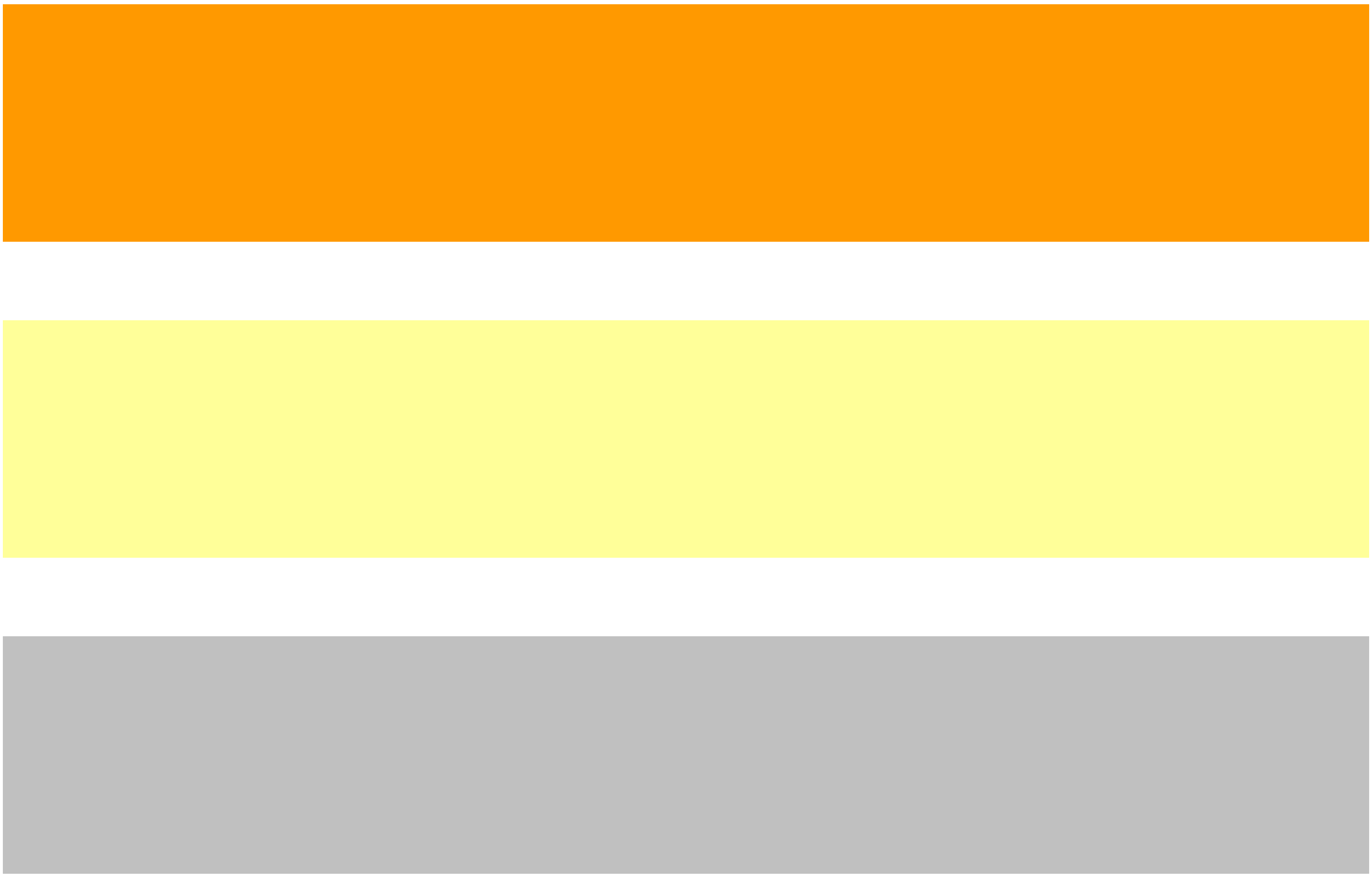

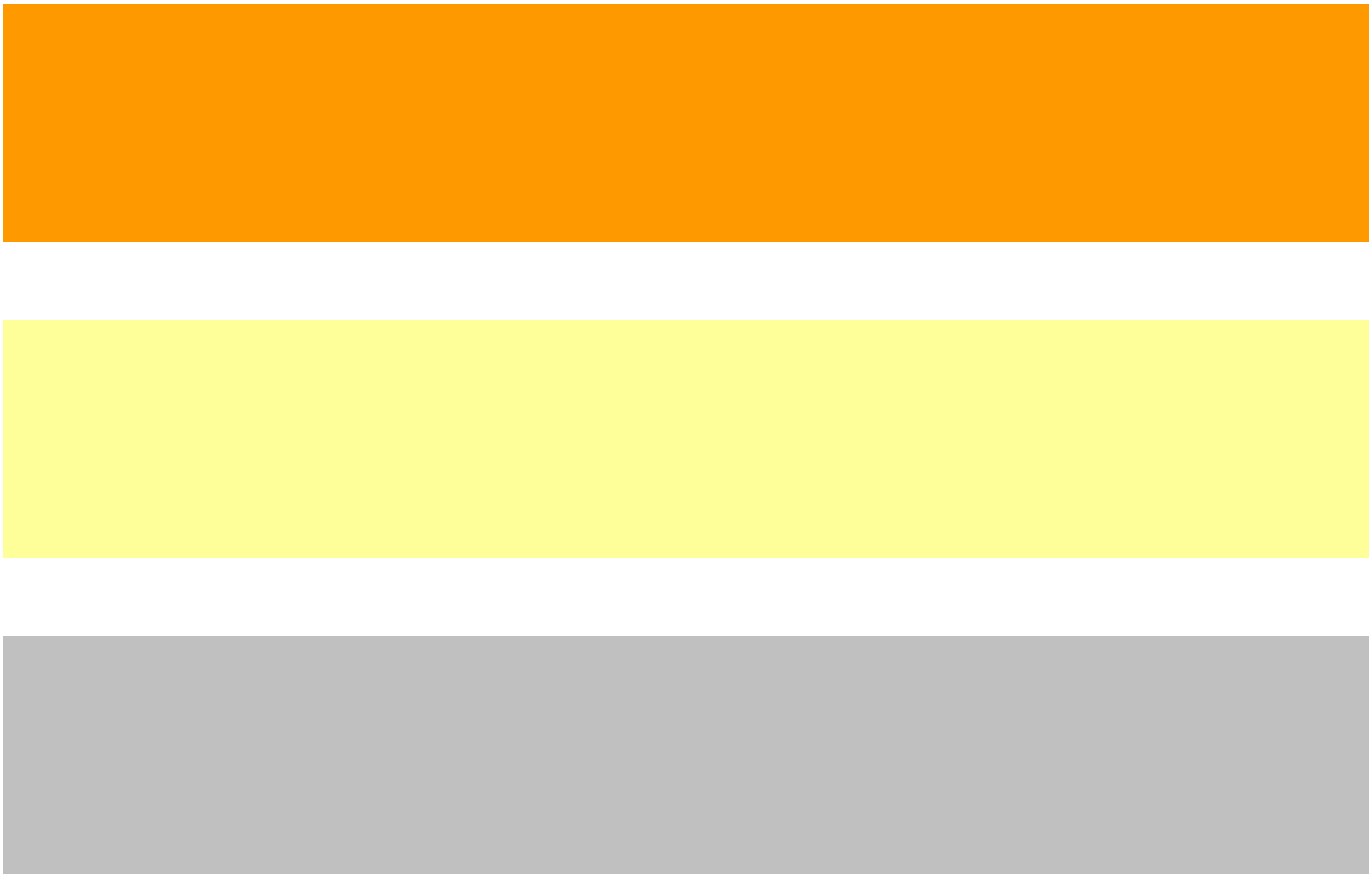

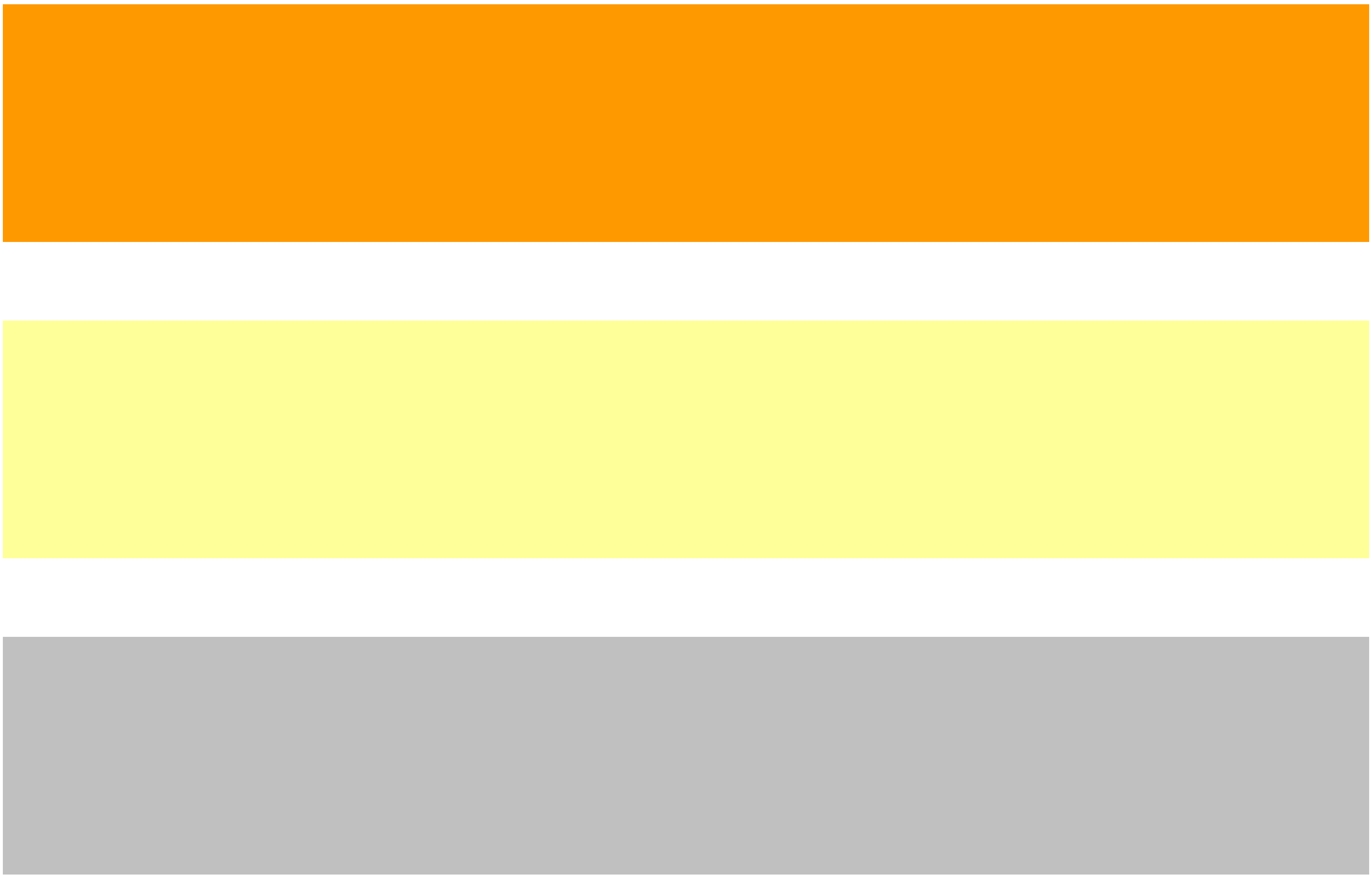

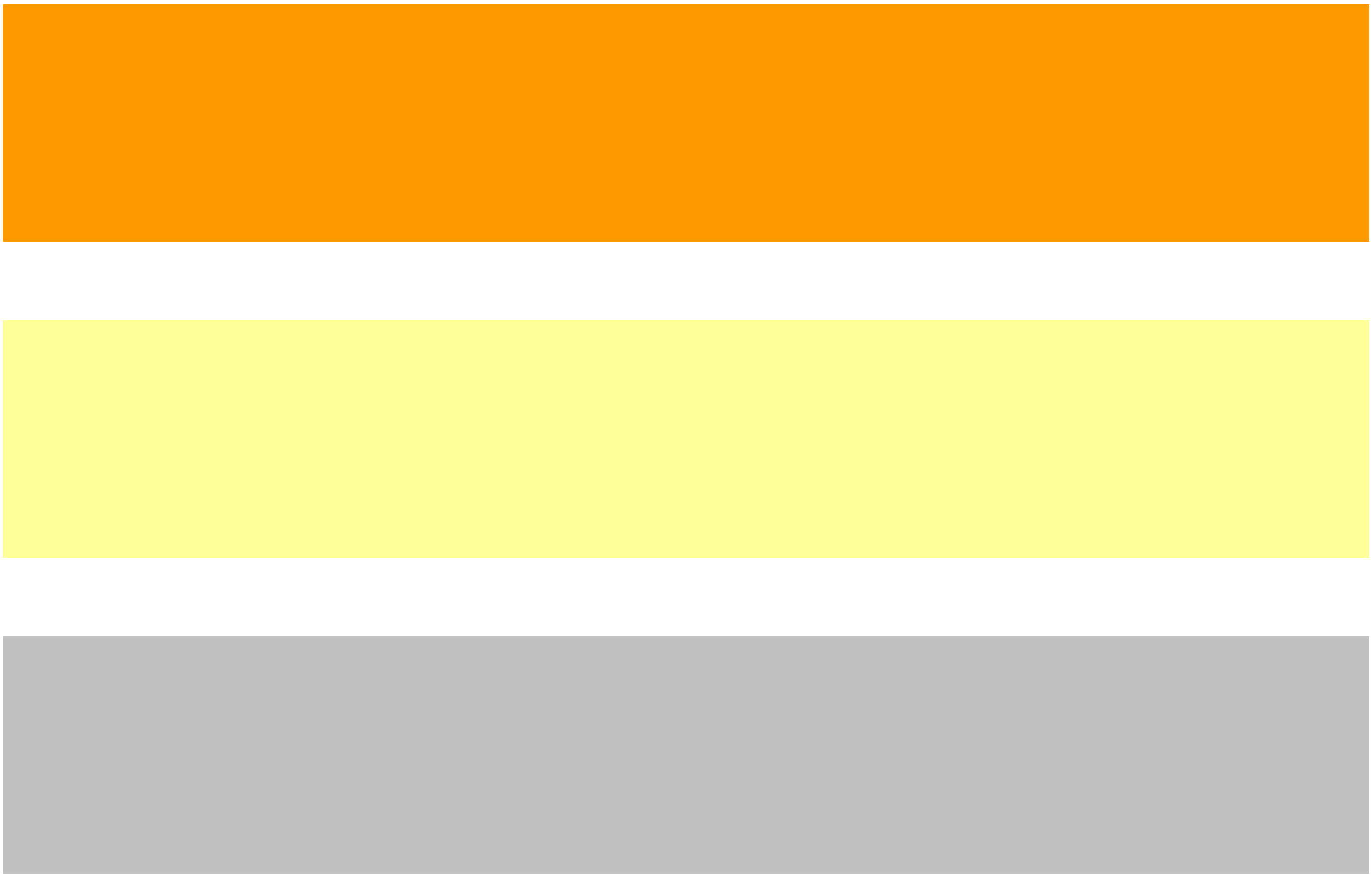

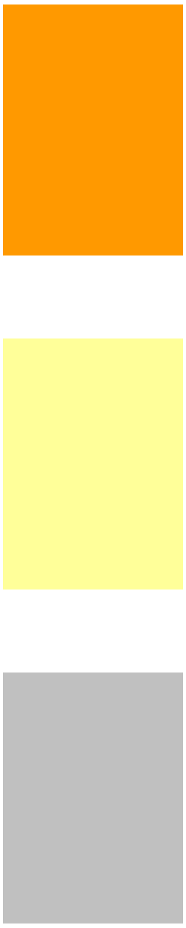

Supplement: S4 File — Micronuclei (BNMN) and CBPI index expression in RAW 264.7 cells treated with particles from truck tires. (PDF) [file pone.0222044.s004.pdf]
