# Supplementary material for: Exposure to particle debris generated from passenger and truck tires induces different genotoxicity and inflammatory responses in the RAW 264.7 cell line
Source: PLoS One. 2019 Sep 10;14(9):e0222044. doi: 10.1371/journal.pone.0222044 (PMC6736306; doi:10.1371/journal.pone.0222044)
Supplement: S6 File — TNF-α release in RAW 264.7 cells treated with truck tires. (PDF) [file pone.0222044.s006.pdf]

TNFa TEST Truck
FOR EACH CONDITION THE SAMPLES HAVE BEEN MEDIATED

4 h lecture

CONDITIONS	ctrl	10 µg/mL	25 µg/mL	50 µg/mL	100 µg/mL	LPS (0,1µg/mL)
	0.124	0.134	0.189	0.206	0.179	0.316
	0.114	0.136	0.190	0.217	0.187	0.302
	0.125	0.150	0.193	0.210	0.190	0.309
MEAN	0.121	0.140	0.191	0.211	0.185	0.309
	0.140	0.134	0.191	0.173	0.221	0.286
	0.146	0.142	0.173	0.196	0.237	0.294
	0.161	0.159	0.191	0.196	0.226	0.320
MEAN	0.149	0.145	0.185	0.188	0.228	0.300
	0.141	0.172	0.179	0.097	0.249	0.238
	0.126	0.196	0.203	0.109	0.221	0.204
	0.131	0.187	0.215	0.111	0.235	0.213
MEAN	0.133	0.185	0.199	0.106	0.235	0.218

Standard curve equation used to calculate the pgr x=(y-0,0147)/0,0005

4h (pg)

CONDITIONS	ctrl	10 µg/mL	25 µg/mL	50 µg/mL	100 µg/mL	LPS (0,1µg/mL)
	212.600	250.600	351.933	392.600	341.267	588.600
	268.600	260.600	340.600	347.267	426.600	570.600
	235.933	340.600	368.600	181.933	440.600	407.267
MEAN	239.044	283.933	353.711	307.267	402.822	522.156
SD	28.129	49.329	14.084	110.883	53.766	99.903
SE	16.240	28.480	8.132	64.019	31.042	57.679

24 h lecture

CONDITIONS	ctrl	10 µg/mL	25 µg/mL	50 µg/mL	100 µg/mL	LPS (0,1mg/ml)
	0.149	0.232	0.244	0.371	0.192	0.450
	0.143	0.241	0.265	0.283	0.209	0.401
	0.116	0.255	0.259	0.320	0.189	0.422
MEAN	0.136	0.243	0.256	0.325	0.197	0.424
	0.119	0.236	0.248	0.322	0.241	0.452
	0.113	0.248	0.238	0.335	0.232	0.419
	0.099	0.252	0.243	0.364	0.221	0.409
MEAN	0.110	0.245	0.243	0.340	0.231	0.427
	0.132	0.328	0.221	0.333	0.235	0.231
	0.142	0.302	0.213	0.323	0.265	0.267
	0.170	0.331	0.206	0.354	0.302	0.234
MEAN	0.126	0.277	0.230	0.339	0.247	0.348

24h (pg)

CONDITIONS	ctrl	10 µg/mL	25 µg/mL	50 µg/mL	100 µg/mL	LPS (0,1mg/ml)
	242.600	455.933	482.600	619.933	363.933	819.267
	191.267	461.267	456.600	651.267	433.267	823.933
	223.552	525.552	431.171	648.124	464.124	667.362
MEAN	219.140	480.917	456.790	639.775	420.441	770.187
SD	25.950	38.747	25.715	17.255	51.312	89.080
SE	14.982	22.371	14.846	9.962	29.625	51.430

DATA HISTOGRAM

CONDITIONS	ctrl	10 µg/mL	25 µg/mL	50 µg/mL	100 µg/mL	LPS (0,1µg/mL)
4h	239.044	283.933	353.711	307.267	402.822	522.156
24h	219.140	480.917	456.790	639.775	420.441	770.187
ES 4h	16.240	28.480	8.132	64.019	31.042	57.679
ES 24h	14.982	22.371	14.846	9.962	29.625	51.430
